# Supplementary material for: Structural basis for the modulation of MRP2 activity by phosphorylation and drugs
Source: Nat Commun. 2024 Mar 4;15:1983. doi: 10.1038/s41467-024-46392-8 (PMC10912322; doi:10.1038/s41467-024-46392-8)
Supplement: Supplementary file 1 — Supplementary Information [file 41467_2024_46392_MOESM1_ESM.pdf]

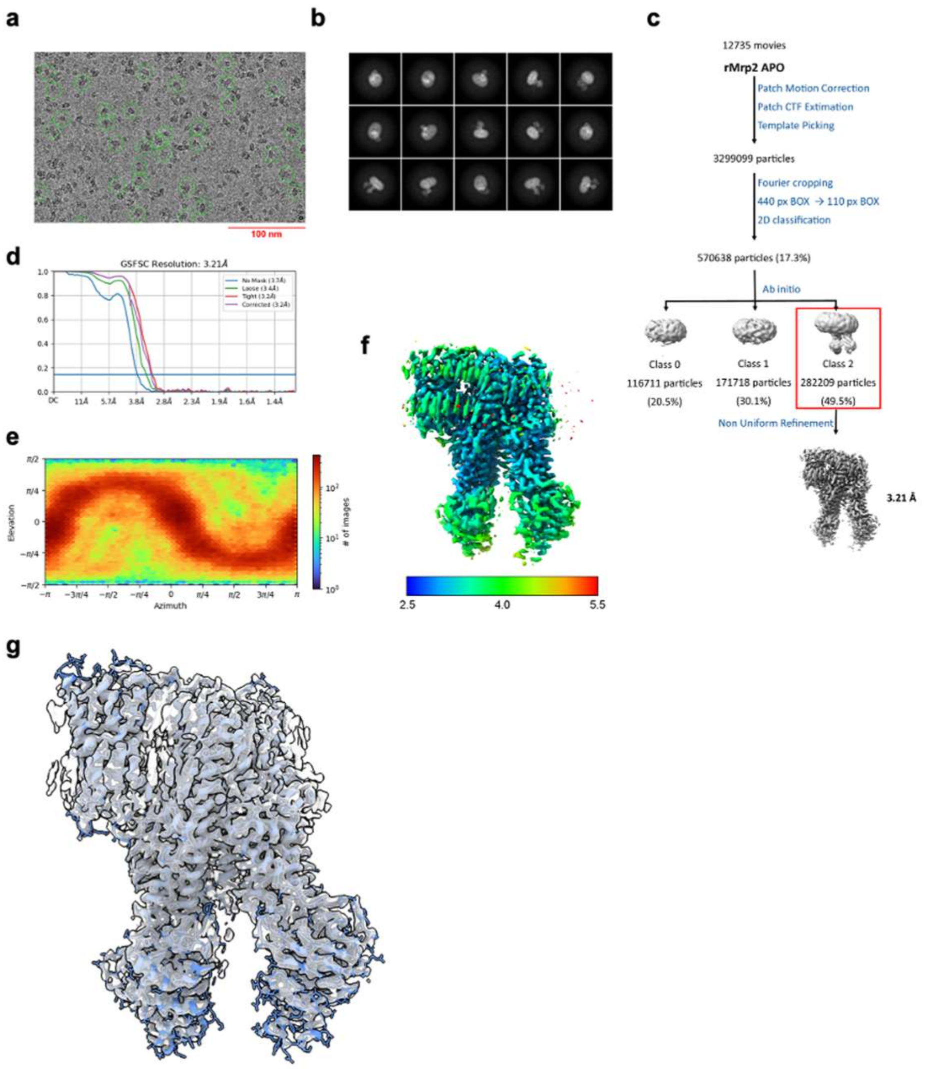

Supplementary Fig. 1. Electron microscopy analysis of nucleotide-free rMrp2. (a) representative electron micrograph (dose-weighted averaged movie) of rMrp2 (individual particles are marked by green circles). (b) A selection of representative 2D class-averages. (c) Data processing pipeline. Blue fonts indicate steps performed in cryoSPARC. (d) GSFSC curve calculated using two independent half-maps (0.143). (e) Euler angle distribution plot. (f) Cryo-EM map of nucleotide-free rMrp2 coloured by local resolution. The resolution for the rMrp2 ranges from ~2.5 to 2.8 Å for the TMD and TMD0, and ~3.0 to 4.0 Å for the NBDs. (g) Representative map for the whole of rMRP2.

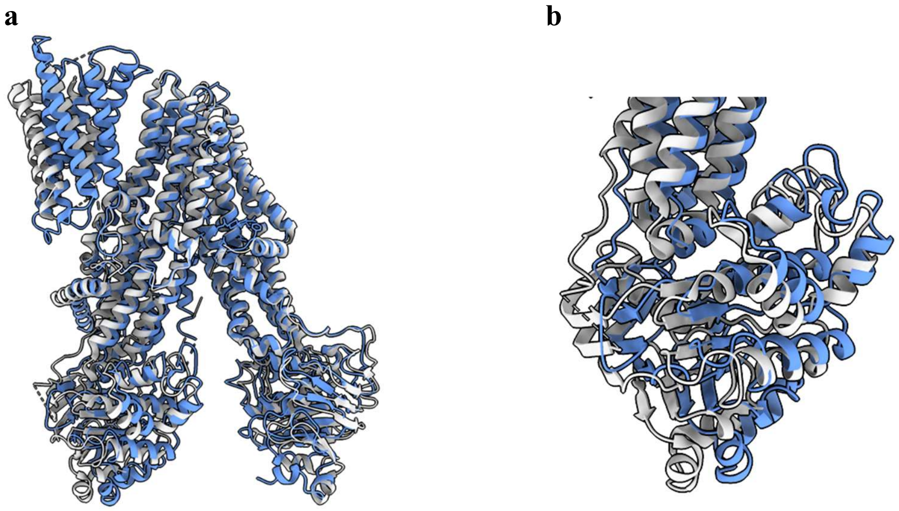

Supplementary Fig. 2. Comparison of the nucleotide-free rMrp2 and bMRP1 structures. (a) rMrp2 (blue) can be superimposed onto the bMRP1 (grey) with an rmsd of 5.6 Å as result of the TMD0 tilt. rMrp2 displays a more closed conformation relative to the bMRP1 due to a 2.5 Å shift towards NBD2. (b) Close-up of the NBD1 and ICL4 interface.

MDKFCNSTFWDLSLLESPEADLPLCFEQTVLVWIPLGFLWLLAPWQLYSVYRSRTKR**SSITKFYLAK**
QVFVVFLLILAAIDLSLALTEDTGQATVPPVRYTNPILYLCTWLLVLAVQHSRQWCVRKNSWFLSLF
WILSVLCGVFQFQTLIRALLKDSKSNMAYSYLFFVSYGFQIVLLILTAFSGPSDSTQTPSVTASFLSSIT
FSWYDR**TVLKGYKHPLTLEDVWDIDEGFKTRSVTSKFEAAMTKDLQ**KARQAFQRRLQKS**QRKPEA**
TLHGLNKKQSQSQDVLVLEEAKKKSE**KTTKDYPKSWLIK**SLFK**TFHVVILKSFILKLIHDLLVFLNPQ**
LLKLLIGFVKSSNSYVWFGYICAILMFAVTLIQSFCLQSYFQHCFVLGMCVR**TTVMSSIYKKALTLSNL**
ARKQYTIGETVNLMSVDSQKLMDATNYMQLVWSSVIQITLSIFFLWR**ELGPSILAGVGVMVLLIPVNG**
VLATKIRNIQVQNMKNKDKRLKIMNEILSGIKILKYFAWEPSFQEQVQGIRKKELKNLLRFGQLQSLLI
FILQITPILVSVVTFSVYVLVDSANVLNAEKAFTSITLFNILRFPLSMLPMVTSSILQASVSVDRLERYL
GGDDLDTSAIRRVSNFDKAVKFSEASFTWDPDLEATIQDVNLDIKPGQLVAVVGTVGSGKSSLVSA
MLGEMENVHGHITIQGSTAYVPQQSWIQNGTIKDNILFGSEYNEKKYQQVLKACALLPDLEILPGGD
MAEIGEKGINLSGGQKQRVSLARAAYQDADIYILDDPLSAVDAHVGKHIFNKVVGPNGLLAGKTRIF
VTHGIHFLPQVDEIVVLGKGTILEKGSYRDLLDKKGVFARNWK**TFMKHSGPEGEATVNNDSEAEDD**
DDGLIPTMEEIPEDAASLAMRRENSLRRTLSRSSRSSSRR**GKSLKNSLKIKNVNVLKEKEKEVEGQ**
KLIK**KEFVETGK**VKFSIYLKYLQAVGWWSILFIILFYGLNNVAFIGSNLWLSAWTSDSDNLNGTNNSSS
HR**DMRIGVFGALGLAQGICLLISTLWSIYACRNASKALHGQLLTNILRAPMRFFDTTPTGRIVNRFSG**
DISTVDDLLPQTLRSWMMCFFGIAGTLVMICMATPVFAIIIIPLSILYISVQVFYVATSRQLR**RLDSVTKS**
PIYSHFSETVTGLPIIRAFEHQQRFLAWNEKQIDINQKCVFSWITSNRWLAIRLELVGNLVVFCSALLL
VIYRKTLTGDVVGFVLSNALNITQTLNWLVRMTSEAETNIVAVERISEYINVENEAPWVTDKRPPAD
WPRHGEIQFNNYQVRYRPELDLVLKGITCNIKSGEKVGVVGR**TGAGKSSLTNCLFRILESAGGQIIID**
GIDVASIGLHDLRERLTIIPQDPILFSGSLRMNLDPFNKYSDEEVWRALELAHLRSFVSGLQLGLLSE
VTEGGDNLSIGQRQLLCLGRAVLRKSK**ILVLDEATAAVDLETDSLIQTTIRKEFSQCTVITIAHRLHTIM**
DSDKIMVLDNGKIVEYGSPEELLSNRGSFYLMAKEAGIENVNHTEL

Supplementary Fig. 3. Mass spectrometry analysis of rMrp2. Peptide coverage, 76%, mapped onto the rMrp2 sequence (UniProt Q63120) after trypsin digest (highlighted in blue and bold). The R-domain residues, G863-T958, are underlined.

a

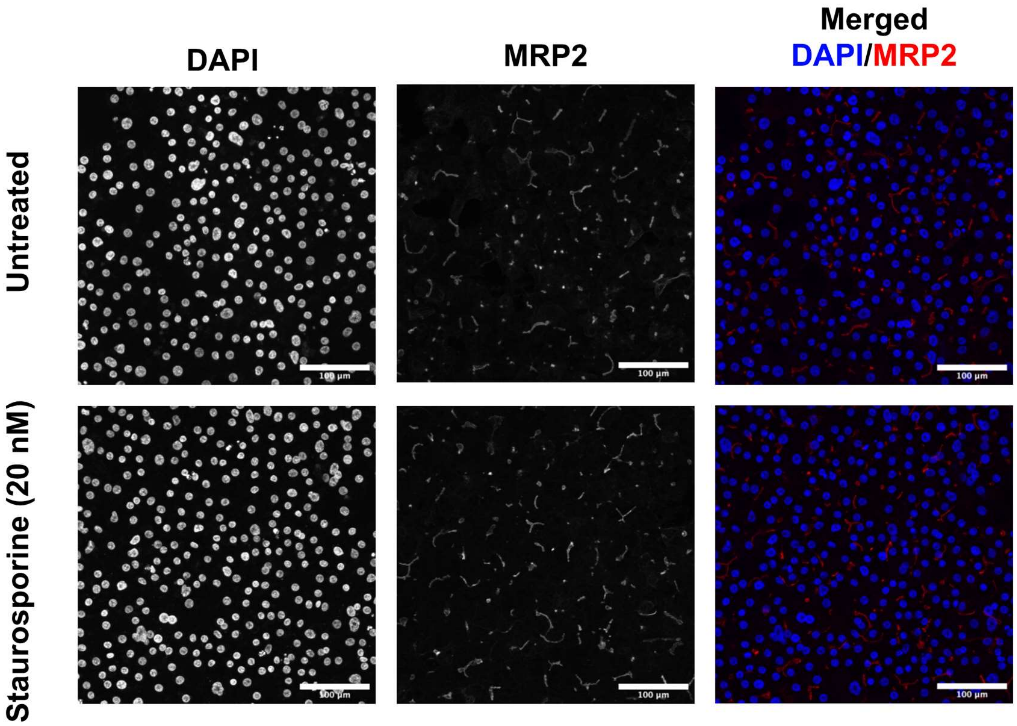

b

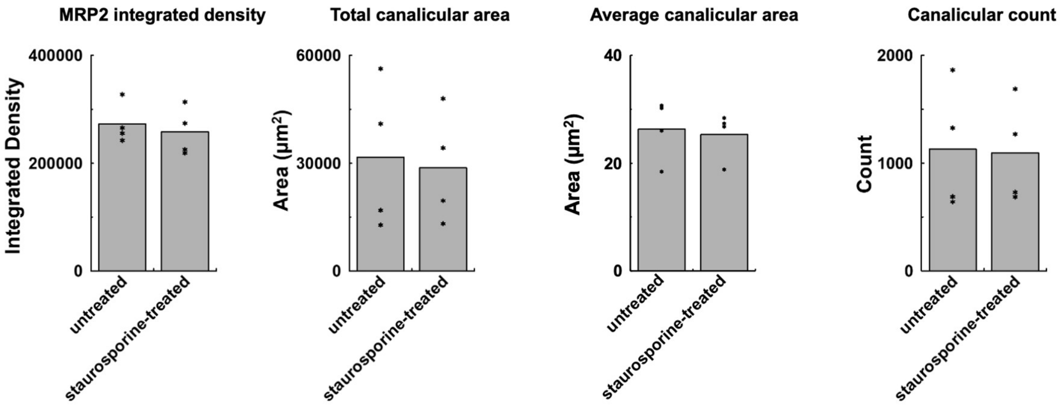

Supplementary Fig. 4. Analysis of human MRP2 localisation and expression upon staurosporine treatment. (a) Live cell imaging of MRP2 (red) expression in the bile canaliculi of iHEPs. Nuclei are stained with DAPI (blue). Staurosporine treatment did not reduce the MRP2 expression or affect its localisation at the canalicular membrane, and it did not disrupt the canalicular structure. Single grayscale images and merged multicolour images are shown. (b) Integrated density of MRP2 expression/localisation, total canalicular area, average canalicular area and canalicular count. Results are represented as means with individual data points indicated by *, from 4 iHEP batches (n=3 images per condition per batch). Not significantly different as estimated by one-tail paired t-test($p=0.32$, $p=0.41$, $p=0.39$, $p=0.46$ respectively).

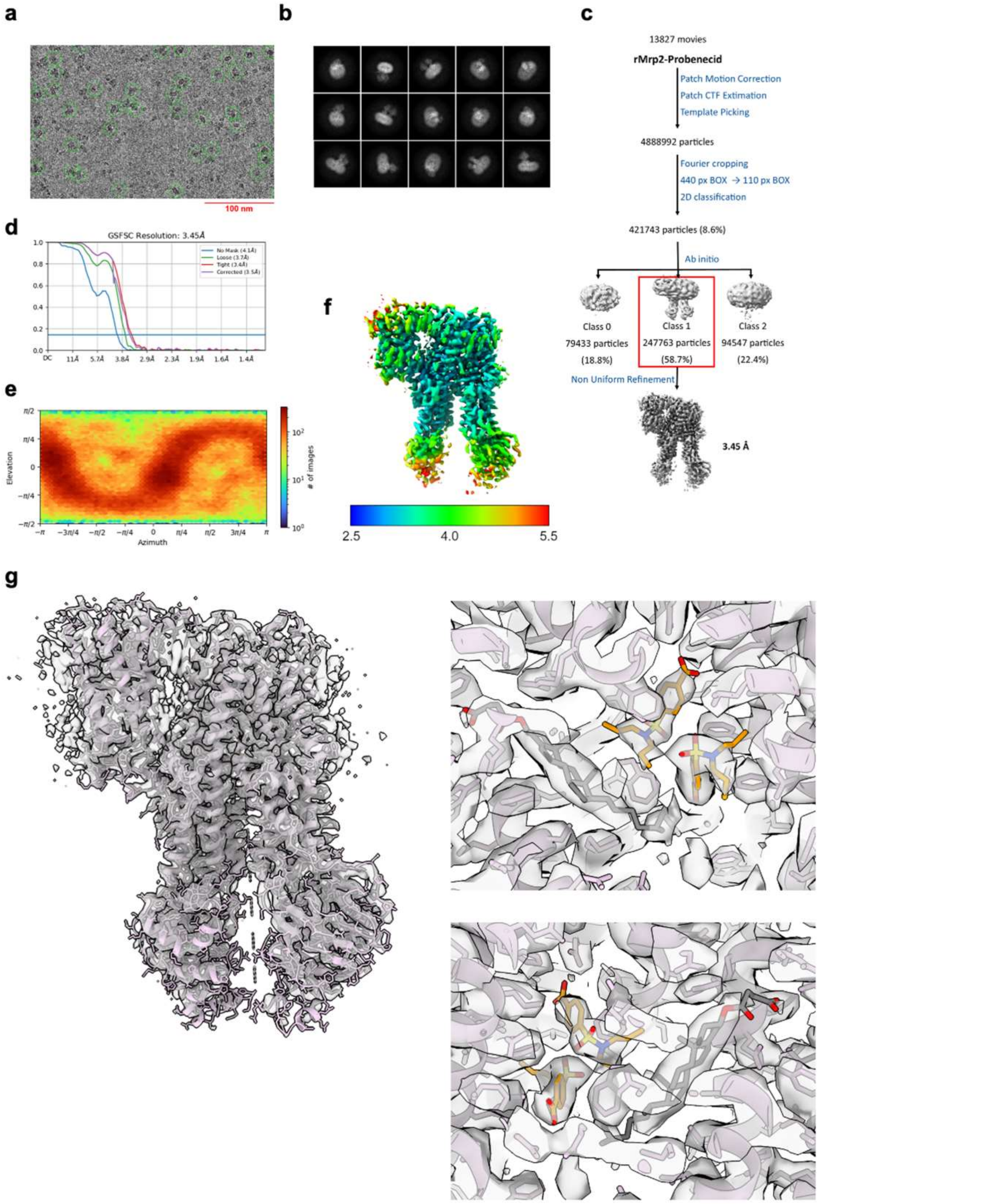

Supplementary Fig. 5. Electron microscopy analysis of rMrp2 in complex with probenecid. (a) representative electron micrograph (dose-weighted averaged movie) of rMrp2 with probenecid (individual particles are marked by green circles). (b) A selection of representative 2D class-averages. (c) Data processing pipeline. Blue fonts indicate steps performed in cryoSPARC. (d) GSFSC curve calculated using two independent half-maps (0.143). (e) Euler angle distribution plot. (f) Cryo-EM map of rMrp2 in complex with probenecid coloured by local resolution. The resolution of the map ranges from ~2.8 to 3.5 Å for the TMD and ~3.0 to 4.0 Å for the NBDs and TMD0. (g) Representative maps: (left panel) whole of rMRP2 bound to probenecid and (right panel) two different views of the drug binding site. Probenecid and CHS are shown in sticks.

Supplementary Table 1. Cryo-EM data collection, refinement and validation statistics

	nucleotide-free autoinhibited rMRP2 (EMDB-19431) (PDB 8RQ3)	*probenecid-bound rMRP2* (EMDB-19433) (PDB 8RQ4)
**Data collection and processing**		
Magnification	130000	130000
Voltage (kV)	300	300
Electron exposure (e–/Å^2)	49.8	52.8
Defocus range (μm)	1.2 – 2.4	1.2 – 2.4
Pixel size (Å)	0.645	0.65
Symmetry imposed	C1	C1
Initial particle images (no.)	3299099	4888992
Final particle images (no.)	282209	247763
Map resolution (Å)	3.21	3.45
FSC threshold	0.143	0.143
Map resolution range (Å)	2.5 – 4.0	2.8 – 4.0
**Refinement**		
Initial model used (PDB code)		
Model resolution (Å)	3.21	3.45
FSC threshold	0.143	0.143
Model resolution range (Å)	2.5 – 4.0	2.8 – 4.0
Map sharpening *B* factor (Å^2)	-127	-114
Model composition		
Non-hydrogen atoms	1432	1405
Protein residues	1432	1402
Ligands		1 CHS and 2 PRB
B factors (Å^2)		
Protein	179	115
Ligand		36.5 (CHS) and 44.1 (PRB)
R.m.s. deviations		
Bond lengths (Å)	0.009	0.006
Bond angles (°)	0.781	0.653
Validation		
MolProbity score	1.91	1.85
Clashscore	9.5	10.5
Poor rotamers (%)	0	0.64
Ramachandran plot		
Favored (%)	93.8	95.5
Allowed (%)	6.2	4.5
Disallowed (%)		
