# Supplementary material for: Structural basis for the modulation of MRP2 activity by phosphorylation and drugs
Source: Nat Commun. 2024 Mar 4;15:1983. doi: 10.1038/s41467-024-46392-8 (PMC10912322; doi:10.1038/s41467-024-46392-8)
Supplement: Supplementary file 3 — Reporting Summary [file 41467_2024_46392_MOESM3_ESM.pdf]

nature portfolio

Corresponding author(s): Konstantinos Beis

Last updated by author(s): Sep 27, 2023

Reporting Summary

Nature Portfolio wishes to improve the reproducibility of the work that we publish. This form provides structure for consistency and transparency in reporting. For further information on Nature Portfolio policies, see our Editorial Policies and the Editorial Policy Checklist.

Statistics

For all statistical analyses, confirm that the following items are present in the figure legend, table legend, main text, or Methods section.

n/a	Confirmed	
☐	☒	The exact sample size (*n*) for each experimental group/condition, given as a discrete number and unit of measurement
☐	☒	A statement on whether measurements were taken from distinct samples or whether the same sample was measured repeatedly
☐	☒	The statistical test(s) used AND whether they are one- or two-sided<br>*Only common tests should be described solely by name; describe more complex techniques in the Methods section.*
☒	☐	A description of all covariates tested
☒	☐	A description of any assumptions or corrections, such as tests of normality and adjustment for multiple comparisons
☐	☒	A full description of the statistical parameters including central tendency (e.g. means) or other basic estimates (e.g. regression coefficient) AND variation (e.g. standard deviation) or associated estimates of uncertainty (e.g. confidence intervals)
☐	☒	For null hypothesis testing, the test statistic (e.g. *F*, *t*, *r*) with confidence intervals, effect sizes, degrees of freedom and *P* value noted<br>*Give P values as exact values whenever suitable.*
☒	☐	For Bayesian analysis, information on the choice of priors and Markov chain Monte Carlo settings
☒	☐	For hierarchical and complex designs, identification of the appropriate level for tests and full reporting of outcomes
☒	☐	Estimates of effect sizes (e.g. Cohen's *d*, Pearson's *r*), indicating how they were calculated

Our web collection on statistics for biologists contains articles on many of the points above.

Software and code

Policy information about availability of computer code

Data collection	cryo-EM data collection using EPU (eBIC)
Data analysis	GraFit 5.0.13, cryoSPARC (4.2), phenix (1.18.2), COOT (0.9.8.91), Chimera X (1.6), IMP-ptmRS node (2.1) , Minora Feature Detector and Precursor Ions Quantifier node (2.3), MolProbity (4.5.2), Fiji (2.9.0)

For manuscripts utilizing custom algorithms or software that are central to the research but not yet described in published literature, software must be made available to editors and reviewers. We strongly encourage code deposition in a community repository (e.g. GitHub). See the Nature Portfolio guidelines for submitting code & software for further information.

Data

Policy information about availability of data

All manuscripts must include a data availability statement. This statement should provide the following information, where applicable:

- Accession codes, unique identifiers, or web links for publicly available datasets
- A description of any restrictions on data availability
- For clinical datasets or third party data, please ensure that the statement adheres to our policy

The cryo-EM maps have been deposited in the Electron Microscopy Data Bank (EMDB) under accession codes EMD-19431 [https://www.ebi.ac.uk/emdb/EMD-19431] (nucleotide-free autoinhibited rMrp2) and EMD-19433 [https://www.ebi.ac.uk/emdb/EMD-19433] (probenecid bound rMrp2). The structural coordinates have been deposited to the RCSB Protein Data Bank (PDB) under the accession codes 8RQ3 [https://doi.org/10.2210/pdb8RQ3/pdb] (nucleotide-free

autoinhibited rMrp2) and 8RQ4 [https://doi.org/10.2210/pdb8RQ4/pdb] (probenecid bound rMrp2). Raw data for nucleotide-free autoinhibited rMrp2 and probenecid bound rMrp2 were submitted to Electron Microscopy Public Image Archive with IDs EMPIAR-11893 and EMPIAR-11894, respectively. The mass spectrometry proteomics data have been deposited to the ProteomeXchange Consortium via the PRIDE partner repository with the dataset identifier PXD045845 [https://proteomecentral.proteomexchange.org/cgi/GetDataset?ID=PXD045845]. Source data are provided with this paper.

Research involving human participants, their data, or biological material

Policy information about studies with human participants or human data. See also policy information about sex, gender (identity/presentation), and sexual orientation and race, ethnicity and racism.

Reporting on sex and gender	n/a
Reporting on race, ethnicity, or other socially relevant groupings	n/a
Population characteristics	n/a
Recruitment	n/a
Ethics oversight	n/a

Note that full information on the approval of the study protocol must also be provided in the manuscript.

Field-specific reporting

Please select the one below that is the best fit for your research. If you are not sure, read the appropriate sections before making your selection.

☒ Life sciences ☐ Behavioural & social sciences ☐ Ecological, evolutionary & environmental sciences

For a reference copy of the document with all sections, see nature.com/documents/nr-reporting-summary-flat.pdf

Life sciences study design

All studies must disclose on these points even when the disclosure is negative.

Sample size	Sample size was not predetermined. 3 biological repeats for protein based assays and 5 repeats for iHEP assays. These were selected to provide statistical significance.
Data exclusions	All data were included in the analysis.
Replication	Three biological repeats were used for all the ATPase assays in destabiised liposomes and transport assays. Five repeats for iHEP assays. All repeats were successful.
Randomization	Randomization was not possible as each protein was at a different phosphorylation state.
Blinding	Blinding was not possible as each protein was at a different phosphorylation state.

Reporting for specific materials, systems and methods

We require information from authors about some types of materials, experimental systems and methods used in many studies. Here, indicate whether each material, system or method listed is relevant to your study. If you are not sure if a list item applies to your research, read the appropriate section before selecting a response.

Materials & experimental systems

n/a	Involved in the study	
☐	☒	Antibodies
☐	☒	Eukaryotic cell lines
☒	☐	Palaeontology and archaeology
☒	☐	Animals and other organisms
☒	☐	Clinical data
☒	☐	Dual use research of concern
☒	☐	Plants

Methods

n/a	Involved in the study	
☒	☐	ChIP-seq
☒	☐	Flow cytometry
☒	☐	MRI-based neuroimaging

Antibodies

Antibodies used	cells were stained with a 1:25 dilution of human MRP2 primary antibody (Abcam; ab3373). The following day, the cells were stained with a 1:500 dilution of AlexaFluor 555 donkey anti-mouse antibody (Invitrogen; A-31570)
Validation	No further authentication was conducted for commercially available antibodies

Eukaryotic cell lines

Policy information about cell lines and Sex and Gender in Research

Cell line source(s)	human embryonic kidney 293 (HEK293) (ATCC: CRL-1573), Human iPSCs (Cell and Gene Therapy Catapult, CGT-RCiB10)
Authentication	No further authentication was conducted for commercially available cell lines
Mycoplasma contamination	Tested by facility manager; negative result
Commonly misidentified lines (See ICLAC register)	No misidentified lines were used
